# Sex-based differences in the NRF2 oxidative stress response: Implications for precision therapeutics

**DOI:** 10.1016/j.redox.2026.104128

**Published:** 2026-03-14

**Authors:** Giusy Russomanno, Karolina Kwiatkowska, Ian M. Copple

**Affiliations:** Department of Pharmacology and Therapeutics, Institute of Systems, Molecular and Integrative Biology, University of Liverpool, Liverpool, L69 3GE, UK

**Keywords:** NRF2 pathway, Sex dimorphism, Drug response, Personalised therapy

## Abstract

Nuclear factor erythroid 2–related factor 2 (NRF2) is a central regulator of antioxidant defences, mitochondrial function, and cellular stress responses, making its pharmacological activation a compelling strategy for neurodegenerative, metabolic, and cardiovascular diseases. Emerging evidence reveals that biological sex profoundly shapes NRF2 signalling, influencing basal activity, inducibility, and downstream functional outcomes. Females often exhibit higher NRF2 target gene expression in liver and kidney, whereas males may be more susceptible to oxidative or metabolic stress due to androgen-mediated suppression of the NRF2 pathway. Hormonal status, age, and tissue-specific receptor distribution further modulate these effects, suggesting that therapeutic responses to NRF2 activators are inherently sex-dependent. Pharmacokinetic and pharmacodynamic differences, including CYP3A-mediated metabolism and body composition, may additionally influence systemic exposure and safety profiles. Despite clinical use of NRF2 activators such as dimethyl fumarate and omaveloxolone, sex-stratified data on efficacy, dosing, and safety are scarce. This knowledge gap underscores the need for systematic evaluation of sex, hormonal milieu, and age in pharmacokinetic, pharmacodynamic, and clinical studies to ensure treatments are safe, effective, and equitable. Integrating these variables into research and clinical practice will optimise therapeutic benefits and minimise adverse events by accounting for patient-specific biology.

## Highlights

• Biological sex is a key but overlooked determinant of NRF2 signalling.
• Females show enhanced antioxidant programmes across tissues.
• Sex differences in pharmacokinetics/dynamics shape therapeutic efficacy and safety.
• Sex, hormonal status, and age should guide NRF2 drug development.

## Graphical abstract

**Figure:**
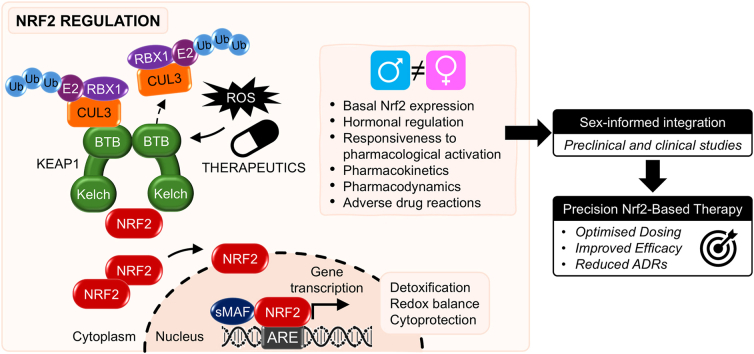

## 1 Introduction

The transcription factor nuclear factor erythroid 2–related factor 2 (NRF2) is a master regulator of the cellular antioxidant response, modulating the expression of a broad range of genes involved in detoxification, redox balance, and cytoprotection [1]. Activation of the NRF2 pathway has been widely explored as a therapeutic strategy across diverse conditions including neurodegeneration, metabolic and cardiovascular diseases, and chronic inflammation [[2], [3], [4], [5], [6]]. Several pharmacological activators of NRF2 have reached the clinical stages of development and use, exemplifying successful translational applications. Dimethyl fumarate (DMF) and omaveloxolone are approved for multiple sclerosis [7] and Friedreich's ataxia [8], respectively, whilst bardoxolone methyl has been evaluated in chronic kidney disease and Alport syndrome [9], demonstrating the pathway's therapeutic potential across diverse diseases.

Although NRF2 plays a central role in mitigating oxidative and electrophilic stress, emerging evidence indicates that its regulation and therapeutic activation may differ between sexes. Studies have shown that certain NRF2 target genes exhibit higher expression in the livers of female mice compared to males, suggesting sex-specific regulatory mechanisms [10]. Additionally, females generally possess more proficient antioxidant defences against reactive oxygen species, which may influence their response to NRF2 activation [11]. These findings underscore the necessity of considering sex differences when evaluating NRF2-targeted therapies. Biological sex has profound effects on immune function, metabolism, hormone signalling, and drug metabolism [12,13] — all of which intersect with NRF2 biology. For instance, sex hormones such as oestrogen and testosterone have been shown to modulate NRF2 expression and activity [14,15], yet these effects remain incompletely understood and are rarely accounted for in therapeutic development or clinical trial design.

From a patient's perspective, the failure to consider sex-based differences in NRF2 pathway activation could adversely impact treatment efficacy, adverse event profiles, and long-term outcomes. Historically, females have been underrepresented in biomedical research, leading to significant gaps in understanding therapeutic responses [16]. Precision medicine initiatives increasingly recognise the importance of sex as a biological variable, yet its integration into NRF2-targeted research and clinical protocols remains limited [17,18].

This review aims to synthesise current evidence on sex-based differences in NRF2 regulation and activation, with a particular focus on the translational implications for patients. We explore both preclinical and clinical studies, highlight areas where sex-specific data are lacking, and discuss how integrating sex differences into research and clinical practice could enhance the safety and efficacy of NRF2-based therapies. For ease of interpretation, we use the capitalised human form of NRF2 and associated genes/proteins throughout this review, despite referring to evidence from other species in many cases.

## 2 NRF2 signalling and its role in health and disease

**Fig. 1:**
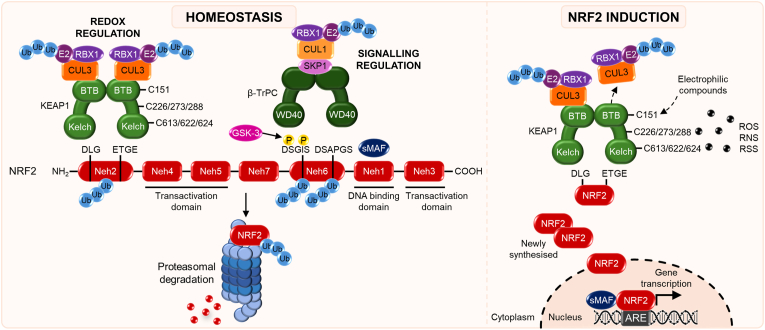
**Overview of the NRF2 signalling pathway.** Under homeostatic conditions, the Kelch domain of KEAP1 binds NRF2 via the DLG and ETGE motifs in its Neh2 domain, enabling CUL3/RBX1-dependent ubiquitylation and subsequent proteasomal degradation. NRF2 ubiquitylation is also controlled by β-TrCP (beta-transducin repeat-containing E3 ubiquitin protein ligase), which recognises the DSGIS and DSAPGS motifs in the Neh6 domain; phosphorylation of the DSGIS motif by glycogen synthase kinase 3 (GSK-3) enhances this interaction. Reactive oxygen (ROS), nitrogen (RNS), and sulphur (RSS) species, as well as electrophilic compounds, modify specific cysteine residues on KEAP1, inducing conformational changes in the CUL3-binding sites in KEAP1 BTB homodimer that weaken KEAP1–CUL3 interactions, thus impairing NRF2 proteasomal degradation. This allows newly synthesised NRF2 to accumulate and translocate to the nucleus to drive the transcription of hundreds of cytoprotective genes. NRF2 transactivation is mediated by the Neh4, Neh5 and Neh3 domains, while Neh1 contains a bZIP motif that binds antioxidant response elements (AREs) during dimerisation with sMAF proteins.

NRF2 serves as a master regulator of cellular defence, preserving homeostasis under oxidative, electrophilic, metabolic, and inflammatory challenges. Under homeostatic conditions, NRF2 is sequestered in the cytoplasm by its negative regulator Kelch-like ECH-associated protein 1 (KEAP1), which acts as a substrate adaptor for the Cullin3 (CUL3)-E3 ubiquitin ligase complex, targeting NRF2 for continuous ubiquitination and proteasomal degradation (Fig. 1) [19]. This tight regulation maintains low basal NRF2 activity. In addition to KEAP1-dependent control, NRF2 can also be degraded via a β-TrCP (β-transducin repeat-containing E3 ubiquitin protein ligase)-mediated pathway, which acts as an auxiliary safeguard mechanism when KEAP1 activity is compromised [20]. Upon exposure to oxidative or electrophilic stresses, reactive cysteine residues on KEAP1 undergo chemical modifications that disrupt KEAP1–CUL3 interactions, stabilising NRF2 and allowing its nuclear accumulation. Specifically, Cys151, Cys273 and Cys288 of KEAP1 act as key sensor residues that are preferentially targeted by electrophiles and oxidants [21,22]. In addition, other cysteines, including Cys226, Cys613, as well as the closely spaced Cys622/Cys624 in the C-terminal region, contribute to redox sensing, particularly under oxidative conditions through disulfide bond formation [23]. Collectively, these modifications induce conformational and functional changes in KEAP1 that impair its ability to act as a CUL3 substrate adaptor and suppress NRF2 ubiquitination. Within the nucleus, NRF2 heterodimerises with small MAF proteins and binds to antioxidant response elements (AREs) in promoter regions of target genes, thereby initiating transcription of a broad cytoprotective programme encompassing antioxidant enzymes, detoxification pathways, and stress-adaptive metabolic reprogramming [6,24,25].

Activated NRF2 regulates several hundred target genes, including classical phase II detoxification enzymes (e.g., NQO1, GSTs, UGTs), components of glutathione biosynthesis (GCLC, GCLM), thioredoxin reductase, peroxiredoxins, and heme oxygenase-1 (HO-1) [24,25]. It also upregulates multiple ATP-binding cassette (ABC) transporters, which promote xenobiotic efflux and confer chemoresistance in certain contexts [25,26]. Beyond its canonical antioxidant functions, NRF2 exerts profound effects on intermediary metabolism. It enhances glycolysis and the pentose phosphate pathway, thereby sustaining NADPH generation, promotes glutaminolysis, and supports mitochondrial function via transcriptional regulation of nuclear respiratory factor-1 (NRF1), peroxisome proliferator-activated receptor-γ coactivator 1-α (PGC-1α), and mitochondrial transcription factor A (TFAM) [27]. These activities preserve mitochondrial redox balance, regulate mitophagy, and facilitate biogenesis, collectively maintaining mitochondrial integrity under conditions of bioenergetic stress.

The protective reach of NRF2 spans many organ systems and disease contexts. The liver, a metabolic hub and major site of xenobiotic detoxification, is a key organ where NRF2 exerts profound effects. Hepatic NRF2 activation induces robust expression of antioxidant and phase II detoxification genes, limits lipid peroxidation, and maintains mitochondrial integrity — critical functions in the context of nutrient overload, alcohol consumption, and exposure to hepatotoxins [28]. Both pharmacological activation and genetic enhancement of NRF2 lower hepatic steatosis, dampen inflammatory signalling, and reduce fibrotic remodelling in preclinical models of metabolic dysfunction–associated steatotic liver disease (MASLD); conversely, NRF2 loss accelerates the transition to steatohepatitis and heightens vulnerability to dietary and toxicant-induced liver injury [[29], [30], [31], [32], [33]]. Importantly, NRF2 intersects with key metabolic regulators in the liver such as AMPK, PPARα, and mTOR, reprogramming hepatic metabolism toward enhanced fatty acid oxidation, suppression of de novo lipogenesis, and autophagy-mediated clearance of damaged organelles [[34], [35], [36]]. Beyond its roles in metabolic regulation and cytoprotection, NRF2 also supports liver regenerative responses after injury or resection [37,38]. NRF2 deficiency has been shown to delay hepatocyte proliferation and impair timely regeneration following partial hepatectomy, in part through dysregulated redox imbalance and growth factor signalling, while pharmacological activation of NRF2 enhances functional recovery of the remnant liver in preclinical models [[38], [39], [40]].

NRF2 is also a central regulator of cardiovascular homeostasis. In the endothelium, NRF2 activation preserves nitric oxide bioavailability and endothelial-dependent vasodilation [41,42]. NRF2 also mitigates vascular smooth muscle cell proliferation and phenotypic switching, processes implicated in neointimal hyperplasia and atherosclerosis [41]. In experimental models of atherosclerosis, NRF2 deficiency accelerates lesion formation, inflammation, and lipid accumulation [43]. Beyond vascular function, NRF2 contributes to myocardial resilience by limiting ischemia–reperfusion injury, attenuating maladaptive cardiac hypertrophy, and preserving mitochondrial bioenergetics in cardiomyocytes [44,45].

In the lungs, NRF2 activation is required for maintaining epithelial barrier integrity and limiting emphysematous changes in models of chronic obstructive pulmonary disease (COPD) and acute lung injury [46,47]. In the central nervous system, NRF2 activation protects neurons and glia by enhancing antioxidant capacity, modulating neuroinflammation, and stabilising mitochondrial function, with demonstrated benefits in models of stroke, Parkinson's disease, Alzheimer's disease, multiple sclerosis, amyotrophic lateral sclerosis (ALS), and Friedreich's ataxia [[48], [49], [50]].

NRF2 also influences proteostasis by regulating autophagy-related genes and interacting with the p62/SQSTM1–KEAP1 feedback loop, integrating redox signalling with selective autophagic clearance of damaged proteins and organelles [36,51]. This broader stress-adaptive role positions NRF2 as a central hub connecting redox biology with metabolic and proteostatic resilience.

Although NRF2 activation typically confers cytoprotective effects, its impact is highly context dependent. For instance, while physiological NRF2 activation is tumour-suppressive by limiting oxidative DNA damage and maintaining genomic stability [[52], [53], [54]], persistent hyperactivation, often due to somatic mutations in KEAP1 or NFE2L2, confers selective advantages to cancer cells. These include enhanced proliferation, metabolic rewiring, inhibition of apoptosis, and chemoresistance, features described across lung, liver, and head and neck cancers [[55], [56], [57], [58], [59], [60], [61]]. Moreover, while moderate NRF2 activation in the cardiovascular system has been shown to be protective, sustained hyperactivation may promote lipid accumulation within macrophages and foam cell formation, potentially exacerbating advanced atherosclerotic lesion formation in a sex-dependent manner, most likely by a combination of systemic metabolic and local vascular effects [62].

Given its pivotal role in orchestrating antioxidant, metabolic, and anti-inflammatory defences, pharmacological activation of NRF2 represents a compelling therapeutic strategy with potential applicability across a broad spectrum of acute and chronic diseases, including metabolic liver disorders, cardiovascular disease and neurodegeneration. However, the dual role of NRF2 as both a cytoprotective and oncogenic factor highlights the necessity of disease-specific and context-dependent therapeutic modulation.

## 3 Biological interplay between NRF2 and sex hormones

**Fig. 2:**
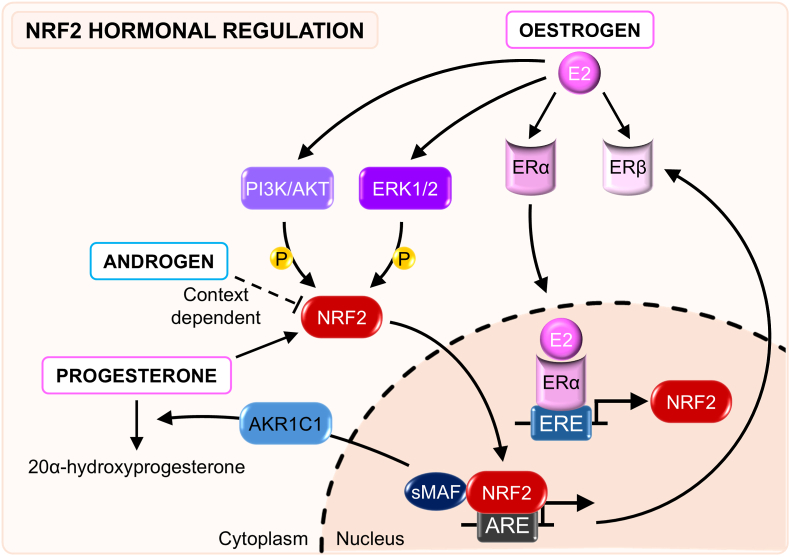
**NRF2 hormonal regulation.** Oestrogens (E2) modulate NRF2 activity through both genomic and non-genomic mechanisms. Upon binding to oestrogen receptors (ERs), E2 enhances NRF2 gene transcription via oestrogen response elements (EREs). E2 can also further promote NRF2 activation through PI3K/AKT and ERK1/2 signalling pathways, leading to increased NRF2 stabilisation and nuclear accumulation. In turn, NRF2 contributes to the regulation of oestrogen signalling by binding antioxidant response elements (AREs) within the ERβ promoter, thereby influencing ERβ expression and supporting a bidirectional regulatory relationship between these pathways. Progesterone has been reported to upregulate NRF2 activity; however, whether this occurs through direct progesterone receptor signalling or indirectly via modulation of upstream redox pathways remains to be clarified. Notably, the NRF2 downstream target AKR1C1 metabolises progesterone to 20α-hydroxyprogesterone, reducing progesterone bioactivity and potentially contributing to resistance to progestin-based therapies. The effects of androgens on NRF2 appear more complex and are highly context-dependent, varying according to tissue type, hormonal concentration, and oxidative status.

Understanding how sex hormones regulate NRF2 is essential for explaining sex-based variation in pathophysiology and for advancing precision therapeutic strategies that target this pathway. The hormonal milieu — fluctuating across the oestrous cycle, menopause, or during menopausal hormone therapy — can modulate NRF2 responsiveness (Fig. 2).

Oestrogens, particularly 17β-oestradiol, have been shown to enhance NRF2 activity in multiple cell types. In neuronal and cardiovascular models, oestrogen increases NRF2 expression and promotes its nuclear accumulation via the PI3K/AKT and MAPK signalling pathways [63,64]. Oestrogen receptor α (ERα)–dependent mechanisms are central to this effect; pharmacological blockade of ERα abolishes oestrogen-induced NRF2 activation in breast cancer cells [14,65]. In addition to ERα-mediated genomic regulation, oestrogen can also elicit rapid, non-genomic effects, including activation of kinases such as PI3K/AKT and ERK1/2 that phosphorylate NRF2, influencing its stability and transcriptional activity [65]. Furthermore, NRF2 itself has been implicated in regulating ERβ expression by binding AREs within the ERβ gene promoter, suggesting a bidirectional regulatory relationship between oestrogen signalling and the NRF2 pathway [66].

Compared to oestrogen, the influence of progesterone on NRF2 signalling has been less extensively characterised. Experimental evidence from models of traumatic brain injury and ischaemic stroke indicates that progesterone can upregulate NRF2 and its downstream targets, contributing to neuroprotection [67,68]. However, whether these effects are mediated directly via progesterone receptors (PR) or indirectly through modulation of upstream redox signalling remains unclear. NRF2 signalling can also impact PR expression and function. In endometrial cancer cells, the overexpression of NRF2 and its downstream target gene AKR1C1 has been associated with resistance to progestin therapy, suggesting that NRF2 activity may modulate PR signalling pathways [55,69].

The relationship between androgens and NRF2 signalling appears complex and context dependent. In models of metabolic syndrome and vascular dysfunction — specifically high-fat-diet-induced obesity in young male mice — testosterone has been reported to suppress NRF2 activity, leading to increased oxidative stress and impaired vascular reactivity [70]. Conversely, in ageing-related neurodegeneration and renal fibrosis, testosterone administration activates the NRF2-ARE pathway, attenuating oxidative damage and improving tissue function [71]. These divergent effects may reflect differences in androgen concentration, tissue-specific distribution of androgen receptors, and the interplay with other signalling pathways, such as NF-κB, though direct mechanistic dissection of these modulators remains relatively unexplored.

Overall, sex hormone-NRF2 crosstalk represent a critical axis in maintaining redox homeostasis and cellular defence, with effects that vary across physiological and pathological contexts.

## 4 Preclinical evidence for sex-based differences in NRF2 activation

Preclinical studies across multiple organ systems demonstrate that sex-based differences in NRF2 activation are both biologically significant and context dependent.

In the liver, baseline rhythmicity and gene expression patterns show clear dimorphism: female mice exhibit consistently higher expression of KEAP1, NQO1, GCLC, and NRF2 throughout the day, reflecting enhanced antioxidant capacity compared with males [72]. Consistent with this, female mice often exhibit higher basal activation of NRF2 target programmes in liver (e.g., NQO1) than males [10,15]. Similar sex-dependent expression of NRF2-regulated genes has also been reported in human liver [73], and experimental models indicate that reduced oestrogen levels, such as following ovariectomy or with ageing, exacerbate metabolic dysfunction when NRF2 activity is compromised [74]. Recent studies in a high-fat diet plus liquid fructose (HFHFr) rat model further highlight sex dimorphism of the KEAP1/NRF2 pathway in response to chronic metabolic challenge [75]. Specifically, males were characterised by an improved ability to cope with HFHFr-induced metabolic stress, primarily through autophagy-mediated proteostatic mechanisms, whereas in female rats this adaptive response appeared impaired. This underscores the complexity of sex-dependent NRF2 regulation, indicating that higher baseline KEAP1/NRF2 expression in females does not necessarily translate into more effective downstream NRF2 functional activity under chronic metabolic challenge, but is shaped by context-specific regulatory crosstalk with adaptive pathways such as autophagy and endoplasmic reticulum stress. In the kidney, female rodents display superior recovery following acute injury, in part through protection against ferroptosis [76,77]. Transcriptomic and single-cell analyses reveal that this sexual dimorphism intersects with NRF2-controlled antioxidant and detoxification pathways, supporting the concept of NRF2 as a contributor to female resilience in renal repair [76,77]. Such differences between males and females may be influenced not only by circulating hormones but also by sex-chromosome-linked epigenetic regulation and broader sex-biased epigenetic mechanisms that impact KEAP1–NRF2 control [78,79].

Sex-specific responsiveness to pharmacological NRF2 activation has also been documented in pre-clinical models. In a keratin-16-null model of palmoplantar keratoderma, the NRF2 activator sulforaphane robustly induced NRF2 activity and prevented disease in male mice, but not in females [80]. In female mice, sulforaphane failed to activate the pathway unless co-administered with an oestrogen receptor β agonist, highlighting the role of sex-specific hormonal modulation of NRF2 responsiveness in skin tissue. Another study in NRF2-deficient mice reported markedly higher sulforaphane tissue concentrations in female null mice compared with males, suggesting that possible sex differences in drug absorption and distribution should be considered when interpreting pharmacological outcomes [81].

In vascular and metabolic contexts, androgen–NRF2 interactions appear to bias outcomes toward males. In young male mice with high-fat diet-induced obesity, testosterone suppresses NRF2 nuclear accumulation and reduces expression of downstream antioxidant enzymes, leading to increased vascular oxidative stress and impaired endothelium-dependent vasodilation [70]. These effects are prevented by castration and reversed by the NRF2 activator bardoxolone methyl [70]. Furthermore, recent work in a Friedreich's ataxia cardiomyopathy mouse model showed that while omaveloxolone broadly improved cardiac function, female mice experienced accelerated mortality compared with males, despite comparable biochemical engagement of NRF2 pathways [82].

Behavioural models also reveal sex-specific effects of NRF2 activation. In a chronic stress paradigm, the electrophilic NRF2 activator DMF produced antidepressant-like effects in male rats but not in females, although both sexes demonstrated improvements in cognitive performance, suggesting that behavioural outcomes resulting from NRF2 modulation may be sex dependent [83]. Additionally, DMF normalised several stress-induced gene expression changes, particularly those related to inflammatory processes in males, whereas in females exhibiting a depressive phenotype a pre-existing reduced inflammatory state was observed.

Although direct, controlled comparisons of NRF2 activation in cultured cells derived from males and females are still lacking, accumulating evidence suggests that cells retain sex-specific differences in oxidative stress responses and antioxidant signalling, supporting the existence of intrinsic, cell-autonomous modulation of redox signalling pathways [84].

Collectively, these findings indicate that sex-based differences in NRF2 activation manifest across a wide range of tissues and experimental contexts. Females often display higher basal or inducible NRF2 activity, particularly in liver and kidney, whereas male-biased suppression of NRF2 is evident under certain metabolic or hormonal conditions. Pharmacological activation of NRF2 may yield sex-dependent outcomes, influenced by factors such as hormonal status, pharmacokinetics, and tissue-specific receptor distribution. These preclinical data emphasise the need for sex to be considered as a biological variable in studies of NRF2 biology and therapeutic targeting.

## 5 Physiological and sex-related influences on drug response

In general, sex-related differences in pharmacokinetics and pharmacodynamics are well established and carry important implications for therapeutic efficacy and safety [85,86]. Beyond body weight, factors such as body fat percentage, total body water, plasma volume, and organ blood flow differ between males and females, and these physiological variations can contribute to sex-specific drug responses [[86], [87], [88]]. Fundamental distinctions in gastrointestinal, hepatic, and renal physiology further influence drug absorption, distribution, metabolism, and elimination [89]. For instance, females generally exhibit lower gastric acid secretion, which may alter drug ionisation and solubility, thereby affecting bioavailability [90]. Sex-related differences in gut microbiota composition also add complexity to gastrointestinal drug handling [[91], [92], [93], [94]]. In addition, the expression of drug transporters is sexually dimorphic: intestinal levels of P-glycoprotein, for instance, are lower in females than in males, potentially modulating absorption and systemic exposure [95].

Distributional differences are also evident; females typically have smaller volumes of distribution for hydrophilic drugs, whereas a higher proportion of body fat increases the distribution of lipophilic compounds, potentially enhancing pharmacological effects or toxicity risks [85]. Sexual dimorphism has been consistently observed for all three major renal functions — glomerular filtration, tubular secretion, and tubular reabsorption — with males generally displaying higher renal clearance than females [96,97]. Significant sex differences have been reported in the expression of hepatic drug-metabolising enzymes. Overall, drugs undergoing biotransformation via phase I and phase II pathways tend to be cleared more rapidly in males than in females [86,98]. By contrast, multiple studies have demonstrated that females exhibit greater cytochrome P450 3A (CYP3A) activity, responsible for the metabolism of over 50% of therapeutics, resulting in approximately 20-30% higher clearance of CYP3A substrates compared to males [86,[99], [100], [101]]. In contrast to CYP3A4, the activities of CYP1A2 and CYP2E1 are higher in males than in females [102].

Importantly, while NRF2 regulates a broad array of detoxification enzymes and antioxidant genes [103,104], drug metabolism and resistance are orchestrated by an integrated network of nuclear receptors and ligand-activated transcription factors, including the aryl hydrocarbon receptor (AhR), the constitutive androstane receptor (CAR), and the pregnane X receptor (PXR), which collectively control the expression of major CYP enzymes and drug transporters [105,106]. Sexual dimorphism has been described not only at the level of CYP enzymes but also in the activity of their upstream regulators. In mouse liver, AhR and AhR-regulated CYP1A genes, as well as CAR mRNA and its target CYP2B10 protein are higher in females compared to males [107]. In addition, PXR has been shown to drive sexually dimorphic hepatic transcriptional responses to gut microbiota signals, influencing xenobiotic and lipid metabolism in a sex-dependent manner [108]. Sex differences in AhR expression have also been observed in rat kidney, pointing to multi-organ dimorphism in xenobiotic receptor regulation [109]. Notably, NRF2 intersects mechanistically with AhR, CAR and PXR signalling pathways through shared response elements and coordinated regulation of phase II enzymes and transporters [103,110]. This interconnected regulatory architecture suggests that sex-based variability in drug response reflects the integrated activity of multiple transcriptional networks rather than NRF2 signalling alone.

Collectively, these findings underscore the clinical relevance of sex differences and may, at least in part, explain variability in safety profiles. Available epidemiological evidence suggests that females are at a substantially elevated risk of adverse drug reactions (ADRs), with incidence rates approximately 1.5- to 1.7-fold higher than in males [[111], [112], [113]]. Furthermore, several studies have reported that women account for around 60% of hospitalisations attributable to ADRs [[114], [115], [116]].

Within this broader pharmacological context, consideration of sex-dependent differences may be particularly relevant for NRF2 activators, especially given the growing clinical interest in their potential to modulate oxidative stress and improve outcomes in a range of chronic and age-related diseases [117]. The biological effects of these compounds are dose- and context-dependent, and many are metabolised via pathways that exhibit sexual dimorphism, raising the possibility that systemic exposure, therapeutic response, and tolerability may differ between males and females.

## 6 Sex differences in clinical responses to NRF2 activators

Clinical experience with NRF2 activators highlights both the therapeutic promise of targeting oxidative stress pathways and the challenges inherent in translating preclinical observations into safe and effective clinical interventions. Bardoxolone methyl (CDDO-Me), a synthetic triterpenoid and potent NRF2 activator, has been evaluated in patients with type 2 diabetes, chronic kidney disease (CKD), and related metabolic disorders [118]. Following oral administration, CDDO-Me undergoes rapid hydrolysis to its active metabolite, 2-cyano-3,12-dioxooleana-1,9-dien-28-oic acid (CDDO), which activates NRF2 by disrupting the KEAP1–NRF2 complex [119]. Pharmacokinetic studies have demonstrated extensive tissue distribution and a long terminal half-life (∼39 h), consistent with prolonged systemic exposure [120]. Early-phase trials reported improvements in estimated glomerular filtration rate (eGFR), raising optimism for its therapeutic potential [118]. However, the Phase III BEACON trial in stage 4 CKD with diabetes was terminated prematurely due to excess adverse events, including heart failure and increased mortality largely attributed to fluid overload and hemodynamic stress [121]. Despite the scale of these studies, sex-stratified analyses of efficacy and adverse outcomes were not reported, leaving open the question of whether males and females respond differently to bardoxolone methyl or experience differential risks.

DMF, an oral NRF2 activator used in relapsing multiple sclerosis, provides a further illustration of the balance between clinical benefit and safety considerations [122,123]. After oral dosing, DMF undergoes rapid esterase-mediated hydrolysis to monomethyl fumarate (MMF), which activates NRF2 and mediates downstream immunomodulatory and cytoprotective effects [124]. MMF displays relatively short systemic exposure (t_max_ 2-4 h; half-life ∼1 h), consistent with minimal accumulation despite twice-daily dosing [124]. In preclinical toxicokinetic studies, female rats exhibited slightly higher systemic exposure (AUC) to MMF compared with males, whereas no significant sex differences were observed in mice, dogs, or cynomolgus monkeys, suggesting species-specific effects [125]. Clinically, DMF effectively reduces relapse activity and radiological inflammation, yet its use is constrained by adverse effects, including gastrointestinal intolerance, flushing, and lymphopenia, which in rare cases predisposes to serious infections [122,123,[126], [127], [128]]. Data from a large cohort study indicate that sex is not a significant risk factor for lymphopenia, suggesting comparable rates in males and females [129]. Although males and females differ in MS susceptibility, relapse rates, and long-term disability trajectories — with females exhibiting greater inflammatory disease activity until menopause and males showing more pronounced neurodegenerative progression [130,131] — there is currently no evidence that DMF efficacy differs by sex [132,133]. Nonetheless, the exclusion of older individuals from clinical trials and the lack of sex-specific analyses limit assessment of potential differences in therapeutic responses or adverse events in postmenopausal females or in males experiencing age-related declines in androgen levels [133]. For instance, menopausal females may be more sensitive to DMF-associated flushing compared to younger females or males.

More recently, omaveloxolone, a semi-synthetic oleanane triterpenoid and potent NRF2 activator, has been approved for the treatment of Friedreich's ataxia (FA) [8,134,135], representing a further clinical application of NRF2 pathway modulation. Omaveloxolone activates NRF2 primarily by inhibiting its KEAP1-mediated degradation, thereby improving mitochondrial function, reducing oxidative stress, and enhancing cellular bioenergetics [136], while also downregulating nuclear factor-κB (NF-κB) signalling and contributing to reduced inflammation [137,138]. Pharmacokinetic analyses indicate that omaveloxolone is orally bioavailable, highly lipophilic, and extensively metabolised by hepatic cytochrome P450 enzymes, with CYP3A playing a major role in its clearance [139,140]. This underpins current dosing recommendations and precautions regarding concomitant use with moderate or strong CYP3A4 inhibitors or inducers. In light of well-established sex differences in CYP3A activity — with females generally exhibiting higher CYP3A-mediated drug metabolism — inter-individual variability in systemic exposure of omaveloxolone may be anticipated [86,[99], [100], [101]], although sex-specific pharmacokinetic analyses have not been formally reported.

In the pivotal MOXIe trial, omaveloxolone demonstrated significant improvements in neurological function, as measured by the modified Friedreich's Ataxia Rating Scale (mFARS), alongside an acceptable tolerability profile [[141], [142], [143], [144], [145]]. Subgroup analyses suggested broadly consistent treatment effects across demographic strata, including sex, while noting that these analyses were exploratory and not powered to detect sex-dependent differences. Omaveloxolone safety profile was characterised primarily by transient elevations in hepatic transaminases, headache, fatigue, and gastrointestinal symptoms [144,146]. However, adverse events have not been systematically reported by sex, precluding definitive conclusions regarding differential susceptibility in males and females. As with other NRF2 activators, the absence of sex-stratified pharmacokinetic, efficacy, and safety analyses limits insight into whether biological sex modifies therapeutic responses or risk profiles, a particularly relevant issue in FA, where disease progression, mitochondrial dysfunction, and hormonal influences may intersect with NRF2-regulated metabolic pathways [[147], [148], [149], [150]].

These findings underscore the potential for sex-specific risk-benefit profiles with NRF2 activation, highlighting the need for future trials to explicitly evaluate sex as a biological variable.

## 7 Conclusions and future directions

Cumulative preclinical and clinical evidence highlights NRF2 activation as a promising therapeutic strategy across multiple organ systems, yet sex-specific differences in the regulation and downstream biological effects of the pathway, along with pharmacokinetics and pharmacodynamics of NRF2 activators, remain underexplored. Preclinical evidence indicates that females often exhibit higher basal or inducible NRF2 activity in liver and kidney, while males may be more susceptible to metabolic or vascular perturbations due to androgen-mediated suppression of NRF2. These differences are influenced by hormonal status, age, and tissue-specific receptor expression, suggesting that both intrinsic biological sex and extrinsic factors such as menopausal status or exogenous hormone therapy could meaningfully modify NRF2-targeted treatment outcomes. Pharmacokinetic and pharmacodynamic studies further underscore sex-dependent variability in drug absorption, distribution, metabolism, and elimination, particularly for compounds metabolised via CYP3A, which could lead to differences in systemic exposure and safety profiles.

Beyond the NRF2 pathway, numerous drug targets and biological pathways exhibit clinically relevant sex differences that influence drug efficacy, safety and dosing [151]. A well-established example is cardiac ion channel signalling, where females have a longer baseline QT interval and a significantly higher risk of drug-induced QT prolongation and torsades de pointes than males for the same exposure to several anti-arrhythmic and psychotropic drugs, reflecting sex-dependent differences in cardiac repolarisation mechanisms [[152], [153], [154], [155]]. Similarly, drug transporters such as P-glycoprotein (ABCB1) show sex-dependent expression and function, with evidence that sex steroids modulate ABC transporter levels and influence drug resistance and disposition across tissues such as the blood‒brain barrier and intestinal epithelium, potentially affecting systemic and central nervous system drug exposure in males versus females [156,157]. Moreover, broad analyses of FDA-approved drug target genes reveal that a substantial proportion show sex-biased expression across human tissues, indicating that many molecular targets routinely engaged by therapeutic agents may inherently differ between males and females, with implications for sex-specific drug action and adverse events [158]. In light of this growing body of evidence, regulatory frameworks are increasingly recognising sex as a critical biological variable in both preclinical and clinical research, moving away from the one-size-fits-all approach, with major funding bodies and agencies now encouraging or mandating the inclusion and analysis of both sexes in study design [[159], [160], [161]]. However, even in well-designed preclinical studies, comparing male and female responses to NRF2 activators is complicated by sex-specific pharmacokinetics, which can mask intrinsic signalling differences.

Although approved NRF2-targeting therapies such as DMF and omaveloxolone have not been associated with major sex-specific efficacy or safety signals to date, these agents were developed and approved in the context of limited sex-disaggregated analyses, and clinical trials were not specifically powered to detect sex-dependent effects. With an increasing appreciation of sex differences in the basal and inducible activity of the NRF2 pathway, there is a need for comprehensive in vitro and in vivo evaluations that systematically account for biological sex, hormonal status, and age at every stage of NRF2-targeted drug development — from model selection and mechanistic studies to clinical trial design and post-marketing surveillance. Such an approach will help to maximise the precision of NRF2-based interventions, optimising efficacy, minimising adverse events, and enabling safer, more personalised treatments across diverse patient populations.

## Funding sources

This work was supported by the 10.13039/501100000265Medical Research Council through a Senior Non-Clinical Fellowship (MR/X007413/1) awarded to Ian M. Copple.

## CRediT authorship contribution statement

**Giusy Russomanno:** Conceptualization, Writing – original draft, Writing – review & editing. **Karolina Kwiatkowska:** Writing – review & editing. **Ian M. Copple:** Conceptualization, Writing – review & editing.

## Declaration of competing interest

The authors declare the following financial interests/personal relationships which may be considered as potential competing interests: Ian M. Copple reports financial support was provided by Medical Research Council. If there are other authors, they declare that they have no known competing financial interests or personal relationships that could have appeared to influence the work reported in this paper.

## Data Availability

No data was used for the research described in the article.
